# Minimal Residual Disease as a Biological Trait: Rethinking Disease Persistence in Hematologic Malignancies

**DOI:** 10.1111/ejh.70203

**Published:** 2026-04-26

**Authors:** Santino Caserta, Enrica Antonia Martino, Ernesto Vigna, Mamdouh Skafi, Antonella Bruzzese, Nicola Amodio, Eugenio Lucia, Graziella D'Arrigo, Virginia Olivito, Caterina Labanca, Francesco Mendicino, Maria Eugenia Alvaro, Giovanni Tripepi, Fortunato Morabito, Massimo Gentile

**Affiliations:** ^1^ Hematology Unit, Department of Onco‐Hematology, AO of Cosenza Cosenza Italy; ^2^ Emergency and Internal Medicine Department Saint Joseph Hospital East Jerusalem Palestine; ^3^ Department of Experimental and Clinical Medicine University of Catanzaro Catanzaro Italy; ^4^ Istituto di Fisiologia Clinica del CNR di Reggio Calabria, Consiglio Nazionale Delle Ricerche Reggio Calabria Italy; ^5^ AIL Sezione di Cosenza Cosenza Italy; ^6^ Department of Pharmacy, Health and Nutritional Science University of Calabria Rende Italy

**Keywords:** clonal evolution, hematologic malignancies, immune evasion, minimal residual disease, tumor microenvironment

## Abstract

Minimal residual disease (MRD) has emerged as a central biomarker in hematologic malignancies, enabling highly sensitive detection of tumor persistence beyond conventional morphologic assessment and serving as an increasingly important surrogate endpoint in clinical trials. Despite these advances, MRD remains predominantly conceptualized as a quantitative variable reflecting residual tumor burden below assay detection thresholds. While this paradigm has enabled standardization of response criteria and cross‐trial comparisons, it does not fully explain key clinical observations, including heterogeneous outcomes among MRD‐positive patients, durable remissions despite detectable disease, and discordance between molecular and imaging‐based assessments. Here, we propose a conceptual framework in which MRD is redefined as a biologically determined trait—a functional phenotype of persistence—rather than a purely quantitative state. We review the mechanisms that shape this phenotype, including therapy‐driven clonal selection, epigenetic and transcriptional plasticity, metabolic adaptation, immune evasion, and microenvironmental niche protection. These processes collectively define the functional fitness of residual tumor cells and their capacity to survive therapeutic pressure, remain dormant, and ultimately drive relapse. This framework provides a mechanistic explanation for clinically observed phenomena—including molecular–imaging discordance and variable relapse kinetics—arguing that these are not merely technical artifacts but reflect distinct, partially independent biological dimensions of residual disease. We further outline a multidimensional model of MRD integrating molecular, spatial, immune, metabolic, and functional dimensions. Operationally, we define MRD as a biological trait across five interacting axes: (i) clonal fitness, (ii) phenotypic plasticity, (iii) metabolic adaptability, (iv) immune evasion, and (v) microenvironmental dependence. Conceptualizing MRD as a dynamic biological trait offers a more comprehensive and testable model of disease persistence and supports the development of mechanism‐based MRD‐directed therapeutic strategies in hematologic malignancies.

## 1 Introduction

Minimal residual disease (MRD) has become a central component of disease assessment in hematologic malignancies, enabling highly sensitive detection of tumor persistence beyond conventional morphologic evaluation. Across multiple disease entities—including multiple myeloma (MM), acute lymphoblastic leukemia (ALL), acute myeloid leukemia (AML), chronic lymphocytic leukemia (CLL), and aggressive lymphomas such as diffuse large B‐cell lymphoma (DLBCL)—MRD status is consistently associated with clinical outcomes and is increasingly incorporated into response criteria, risk stratification algorithms, and clinical trial endpoints [1, 2, 3, 4, 5, 6, 7, 8, 9, 10].

In MM and acute leukemias, achievement of MRD negativity is strongly associated with improved progression‐free and overall survival, supporting its use as a surrogate endpoint in clinical and regulatory settings [1, 2, 6, 8, 9, 11, 12]. In CLL, MRD has emerged as a key determinant of treatment duration and depth of response in targeted therapy‐based regimens [9].

In aggressive lymphomas such as DLBCL, circulating tumor DNA (ctDNA)‐based MRD assessment has expanded the scope of disease monitoring, enabling highly sensitive detection of residual disease and early identification of relapse, often preceding clinical or radiographic progression by several months [3, 4, 5, 13, 14, 15].

Despite these advances, the prevailing conceptual framework for MRD remains fundamentally rooted in a quantitative paradigm, in which MRD is defined as the residual tumor burden below the limit of detection of available assays [8, 9, 12]. Although this framework has enabled standardization, it provides a probabilistic estimate of relapse risk and does not fully capture the biological determinants of disease persistence.

Importantly, several clinical observations challenge this model, demonstrating that patients with similar MRD levels may experience markedly different outcomes, ranging from rapid relapse to prolonged disease control [1, 6, 8]. A particularly informative example is the frequent discordance between molecular and imaging‐based assessments: ctDNA positivity may occur in patients with complete metabolic response on positron emission tomography, while residual imaging abnormalities may persist in the absence of molecular evidence of disease [6, 14, 16]. Collectively, these findings suggest that MRD may not solely be determined by tumor burden, but reflects multiple orthogonal biological dimensions. Thus, discordance should not be interpreted solely as a limitation of measurement, but rather as evidence that distinct assays capture different aspects of residual disease biology.

Emerging evidence suggests that residual disease is enriched for subpopulations with distinct functional characteristics that confer resistance to therapy and enable long‐term persistence. These include genetically selected clones shaped by clonal evolution [11, 17, 18], as well as cells undergoing adaptive phenotypic transitions driven by epigenetic and transcriptional reprogramming [12, 13, 19].

Residual cells also display metabolic rewiring that enhances survival under stress [20, 21, 22], evade immune surveillance through immunoediting and immune escape mechanisms [23, 24, 25], and exploit protective microenvironmental niches [15, 26, 27, 28, 29]. Advances in single‐cell and spatial profiling technologies have further revealed that MRD represents a complex and dynamic ecosystem shaped by tumor–host interactions [21, 30, 31].

Importantly, the quantitative MRD framework has provided a critical foundation for clinical research and patient management. The biological perspective proposed here should therefore be viewed as a complementary extension aimed at explaining clinical heterogeneity rather than replacing established MRD assessment strategies.

Based on these observations, MRD may be viewed not only as a quantitative measure of tumor burden but also as a biologically determined phenotype reflecting the functional properties of residual tumor cells. In this perspective, MRD represents the capacity of residual cells to survive therapy, persist within protective niches, and ultimately drive relapse.

In the following sections, we outline a conceptual framework in which MRD can be interpreted as a biological “trait” shaped by multiple interacting biological dimensions [8, 32].

This framework contrasts with the traditional “state” model of MRD, in which residual disease is defined quantitatively by assay sensitivity. Whereas the state model is measurement‐driven and static, the trait model is biology‐driven and dynamic.

Operationally, MRD as a trait can be conceptualized across five interacting axes: clonal fitness, phenotypic plasticity, metabolic adaptability, immune evasion, and microenvironmental dependence. These axes collectively determine the persistence and clinical behavior of residual disease and can be linked to emerging measurable biomarkers and technologies that allow multidimensional characterization of residual disease, providing a potential pathway toward clinical implementation.

**FIGURE 1:**
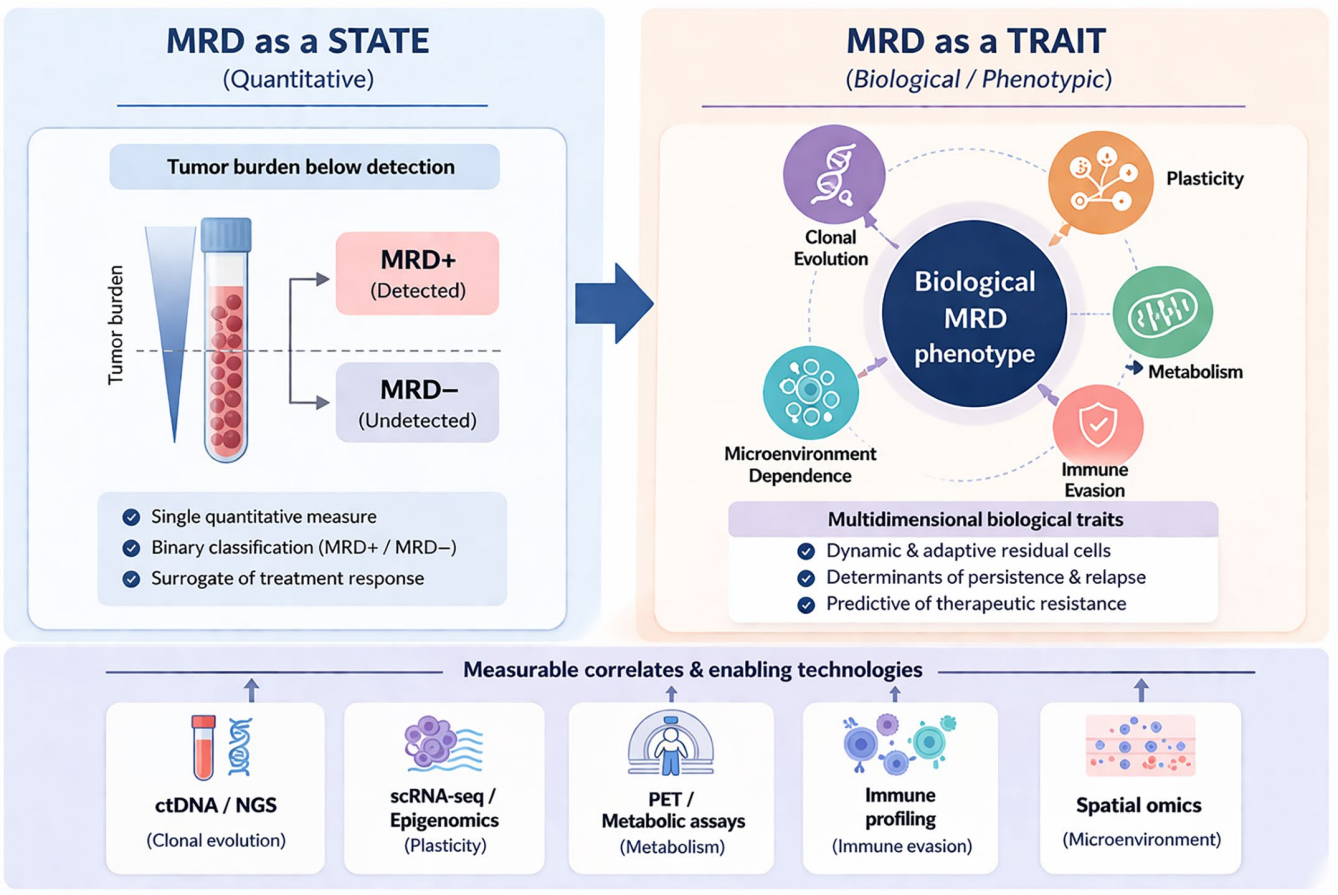
MRD as a state versus MRD as a biological trait. Schematic representation of two conceptual frameworks for minimal residual disease (MRD) in hematologic malignancies. The left panel illustrates the traditional quantitative (state) model, in which MRD is viewed as a function of tumor burden below the limit of detection, leading to a binary classification (MRD‐positive vs. MRD‐negative) and serving primarily as a surrogate marker of treatment response. The right panel depicts the biological (phenotypic) model, in which MRD is conceptualized as a dynamic and adaptive population of residual tumor cells characterized by distinct biological traits, including clonal evolution, cellular plasticity, immune evasion, metabolic adaptation, and microenvironmental niche protection. Importantly, each of these biological dimensions can be associated with measurable correlates and enabling technologies, including circulating tumor DNA (ctDNA) and next‐generation sequencing (NGS) for clonal dynamics, single‐cell transcriptomic and epigenetic profiling for cellular plasticity, metabolic imaging and functional assays for metabolic adaptation, immune profiling for immune escape, and spatially resolved approaches for microenvironmental interactions. This framework emphasizes that MRD reflects not only the quantity of residual cells but also their functional properties and capacity for persistence, thereby providing a more comprehensive operationally measurable framework for understanding relapse risk and therapeutic resistance. Figure generation assisted by AI‐based tools.

Figure 1 summarizes this shift from a purely quantitative to a biologically informed view, contrasting the traditional “state” model with the proposed “trait” framework and introducing the key axes that will be developed in subsequent sections.

## 2 MRD As a State: The Quantitative Paradigm

The conventional framework for MRD is grounded in a quantitative paradigm, in which residual disease is defined as the measurable fraction of malignant cells that persists below the sensitivity threshold of standard morphologic assessment. Within this model, MRD is conceptualized as a state variable, representing tumor burden on a continuous scale that can be operationalized through increasingly sensitive detection technologies.

This paradigm is inherently measurement‐driven. MRD does not exist as an independent biological category, but rather arises from the interaction between tumor biology and the limits of detection of the assay employed. As such, MRD positivity or negativity reflects not only the presence or absence of residual cells but also the sensitivity, specificity, and sampling characteristics of the detection method. Technologies, including multiparameter flow cytometry, PCR‐based approaches, next‐generation sequencing, and ctDNA analysis, have progressed.

Technologies including multiparameter flow cytometry (MFC), PCR‐based approaches, next‐generation sequencing (NGS), and ctDNA analysis have progressively expanded sensitivity and applicability across disease contexts [8, 9, 33]. A key strength of this framework lies in its standardizability, scalability, and clinical applicability. By reducing residual disease to a numerical variable, the quantitative model enables harmonization of response criteria across clinical trials and disease settings and supports the validation of MRD as a surrogate endpoint for clinical outcomes [1, 8, 34]. In this sense, MRD functions as a probabilistic marker of relapse risk, where lower residual burden correlates with improved clinical outcomes at the population level.

However, the quantitative paradigm rests on several implicit assumptions that limit its explanatory power. First, it assumes biological homogeneity among tumor cells, implying that relapse risk is primarily a function of cell number. This *overlooks* the potential for rare, highly adapted subpopulations, or therapy‐resistant subpopulations to drive relapse disproportionately [35]. Second, it treats MRD as a static measurement, typically assessed at discrete timepoints, thereby failing to capture the temporal dynamics of clonal evolution and adaptive responses. Third, it does not account for spatial heterogeneity, as sampling from peripheral blood or bone marrow may not reflect disease localized within protected niches.

More fundamentally, the quantitative model implicitly equates detectability with clinical relevance. Residual populations below detection thresholds may remain biologically competent, while detectable signals may not correspond to viable or proliferative disease. Thus, MRD status reflects a composite of tumor burden, assay characteristics, sampling compartment, and the underlying biology of residual clones and their interactions with host microenvironments.

These limitations underscore that the “state” model provides a probabilistic and measurement‐driven representation of disease persistence, rather than a direct reflection of the biological processes that govern relapse. Table 1 illustrates the conceptual shift required to move from a measurement‐focused assessment toward functional and mechanistic insight. While the quantitative framework remains clinically useful and indispensable for standardization, its limitations motivate the development of complementary, trait‐based MRD approaches that explicitly account for the functional properties of residual clones, their adaptive capacity, and microenvironmental interactions.

**TABLE 1:** Minimal residual disease as a state versus a biological trait: Conceptual and clinical differences.

Feature	MRD as a state (quantitative model)	MRD as a trait (biological model)
Definition	Residual tumor burden below the limit of detection	Biologically defined population of therapy‐persistent cells
Conceptual nature	Static measurement	Dynamic and adaptive phenotype
Primary readout	Binary (MRD‐positive vs MRD‐negative)	Multidimensional (quantity, function, and context)
Key determinant	Number of residual cells	Functional properties and biological fitness
Underlying mechanisms	Not explicitly considered	Clonal evolution, cellular plasticity, metabolic adaptation, immune evasion, microenvironmental interactions
Spatial consideration	Limited or absent	Explicit (niche localization and compartmentalization)
Temporal dimension	Single timepoint assessment	Dynamic evolution over time (kinetics and adaptation)
Clinical interpretation	Prognostic marker of relapse risk	Integrated assessment of relapse potential and biological behavior
Implications for therapy	Guides treatment intensity based on MRD status	Enables mechanism‐based therapeutic targeting (e.g., dormancy, metabolism, immune escape)
Limitations	Ignores heterogeneity and adaptive mechanisms	Requires complex integration and advanced technologies

Despite these conceptual limitations, the quantitative MRD paradigm remains one of the most robust and clinically validated biomarkers in hematologic malignancies. Across multiple diseases, including multiple myeloma, acute lymphoblastic leukemia, and chronic lymphocytic leukemia, MRD negativity has consistently demonstrated strong associations with progression‐free and overall survival and has become an important endpoint in clinical trials and regulatory evaluation.

The strengths of this paradigm lie in its reproducibility, standardization, and scalability across clinical settings. Techniques such as multiparameter flow cytometry, PCR‐based assays, and next‐generation sequencing have enabled increasingly sensitive and harmonized MRD detection, supporting risk stratification and therapeutic decision‐making in routine practice.

Therefore, the goal of the present framework is not to replace the quantitative MRD model but to complement it by incorporating biological dimensions that may explain the heterogeneity observed among patients with similar MRD levels.

## 3 MRD As a Trait: The Biological Paradigm

In contrast to the quantitative framework, an alternative model conceptualizes MRD not as a measurement‐derived state, but as a biologically defined trait—a functional phenotype emerging from the selective pressures imposed by therapy and the tumor microenvironment. Within this paradigm, MRD is not simply the residual fraction of a tumor that escapes detection, but rather a functionally distinct cellular population characterized by specific properties that enable survival, persistence, and eventual re‐expansion.

This distinction reflects a conceptual shift in how residual disease is interpreted. Whereas the “state” model treats MRD as a dependent variable of tumor burden, the “trait” model considers MRD as an emergent property of tumor biology, shaped by evolutionary selection, cellular plasticity, and ecological interactions. In this view, MRD may be influenced not only by how many cells remain but also by the functional properties of those cells. This concept is particularly evident in CLL, where MRD‐positive patients may experience prolonged disease control both during continuous targeted therapy and following fixed‐duration treatment discontinuation, indicating that residual cells may persist in a biologically constrained or therapy‐modulated state rather than uniformly driving relapse [9].

Such observations suggest that the *quality* and context of residual cells, rather than their mere presence, govern clinical trajectories. At the core of this framework lies the concept of selection under therapeutic pressure. Cytotoxic and targeted treatments act as strong evolutionary filters, eliminating sensitive clones while enriching for subpopulations with intrinsic or adaptive resistance. Longitudinal genomic and cytogenetic analyses in hematologic malignancies have demonstrated that relapse is frequently driven by minor subclones present at diagnosis or by populations that acquire resistance‐associated features during therapy [13, 23, 36]. These findings indicate that MRD represents a selected subset of the tumor ecosystem, rather than a random remnant.

In CLL, therapy‐driven clonal evolution is well documented, particularly under Bruton tyrosine kinase inhibition, where resistant subclones harboring BTK or PLCG2 mutations emerge during treatment and may be detectable before clinical progression [24].

Beyond genetic selection, residual cells frequently exploit non‐genetic adaptive mechanisms that confer phenotypic flexibility. Epigenetic remodeling and transcriptional reprogramming, and metabolic adaptation can induce reversible drug‐tolerant states, allowing cells to survive therapy without acquiring stable resistance mutations [19, 25, 37]. Moreover, convergent epigenetic evolution has been documented in AML, where relapse occurs without new driver mutations but with distinct chromatin accessibility signatures, underscoring non‐genetic MRD evolution [26].

These observations challenge the assumption that resistance is solely genetically encoded and instead support a model in which MRD is sustained by dynamic, reversible phenotypic states.

A defining feature of the trait‐based model is the recognition that MRD is embedded within a biological context that critically shapes its behavior. Residual cells interact with the microenvironment, immune system, and metabolic constraints in ways that influence survival and expansion. For example, bone marrow niches in MM and leukemia provide survival signals and protection from therapy, whereas immune evasion mechanisms—such as checkpoint upregulation, impaired antigen presentation, and immunoediting—allow MRD cells to persist despite immune surveillance [23, 24, 25, 26, 27, 28, 29]. These interactions highlight that MRD is not solely a property of tumor cells, but of a tumor–host ecosystem.

Importantly, this framework provides a coherent explanation for clinical phenomena that are difficult to reconcile within the quantitative model. Variability in outcomes among patients with similar MRD levels, as well as discordance between molecular and imaging assessments, can be understood as reflecting differences in the functional competence of residual cells, rather than inconsistencies in measurement alone. In this sense, MRD positivity is not a uniform condition but encompasses a spectrum of biological states with distinct relapse potential.

Conceptualizing MRD as a trait also implies that residual disease is inherently dynamic. Cells may transition between proliferative, quiescent, and drug‐tolerant states in response to environmental cues and therapeutic pressure, altering their sensitivity to treatment over time. This plasticity suggests that MRD should be viewed as a moving target, whose properties evolve throughout the disease course rather than remaining fixed at a given level of tumor burden.

Taken together, the trait‐based paradigm reframes MRD as a functional phenotype of persistence, integrating genetic selection, phenotypic plasticity, microenvironmental influence, and clonal dynamics into a unified model. This perspective complements the quantitative approach but extends beyond it, providing a mechanistic foundation for understanding relapse and a conceptual basis for developing therapies specifically targeting the biological programs that sustain residual disease [8, 32].

## 4 Clonal Selection and Evolution Under Therapy

A central pillar of the “trait” model of MRD is the recognition that residual tumor cells are not a random subset of the diagnostic population, but rather the product of therapy‐driven selection processes. Both cytotoxic and targeted treatments act as potent evolutionary bottlenecks, eliminating sensitive clones while allowing the survival and expansion of subpopulations with intrinsic or adaptive resistance. In this framework, MRD represents the output of Darwinian selection within the tumor ecosystem, enriched for cells with enhanced fitness under therapeutic pressure.

Genomic studies across hematologic malignancies have provided direct evidence for this process. In AML, whole‐genome sequencing of paired diagnosis and relapse samples has demonstrated that relapse frequently arises from minor subclones present at baseline or from ancestral clones that survive therapy and subsequently acquire additional mutations [36]. Similar principles have been observed in lymphoid malignancies. In DLBCL, high‐throughput sequencing and ctDNA‐based monitoring demonstrate that treatment reshapes clonal architecture, with selective outgrowth of resistant subclones and loss of chemosensitive populations [23, 27, 29].

Importantly, clonal selection is not limited to pre‐existing genetic heterogeneity but may also involve adaptive evolutionary trajectories under therapeutic pressure. Longitudinal analyses using ctDNA have revealed dynamic changes in clonal composition during treatment, with early molecular responses followed by the emergence of resistant clones that ultimately drive relapse [13]. These findings highlight that MRD is not a static reservoir but a continuously evolving population, shaped by ongoing selective forces [3, 4]. These findings highlight that MRD is not a static reservoir but a continuously evolving population, shaped by ongoing selective forces.

From a functional perspective, the clones that persist within MRD often display distinct biological properties compared with the bulk tumor. These include activation of survival signaling pathways, resistance to apoptosis, and the ability to withstand genotoxic stress. In MM, for example, residual cells following therapy have been shown to exhibit transcriptional programs associated with stress adaptation and survival, consistent with selection for more resilient phenotypes [6, 30].

A further layer of complexity is introduced by the observation that clonal fitness is context‐dependent. Subclones that are minor or clinically silent at diagnosis may gain a selective advantage under therapy, particularly if they harbor features that confer resistance to specific agents. This phenomenon has been well documented in both hematologic and solid malignancies, where treatment selects for clones carrying alterations in drug targets or downstream signaling pathways [17, 18, 36]. However, even in the absence of clear resistance mutations, subclonal populations may persist through non‐genetic adaptive mechanisms, which interact with genetic selection to shape MRD composition [26, 37].

These insights challenge the implicit assumption of the quantitative MRD model that relapse risk is proportional to the number of residual cells. Instead, they support a model in which relapse is driven by the qualitative properties of selected clones, with a small number of highly adapted cells potentially carrying greater clinical significance than a larger population of less fit cells, consistent with evolutionary models of tumor progression and relapse [17]. In this sense, MRD reflects an evolutionary bottleneck, in which the surviving population is enriched for traits that favor long‐term persistence and eventual regrowth.

Importantly, clonal evolution within MRD is not necessarily associated with high proliferative activity. Residual clones may adopt low‐cycling or quiescent states, allowing them to evade therapies that preferentially target dividing cells while maintaining the capacity for rapid re‐expansion once selective pressures are relieved. This behavior is consistent with experimental evidence demonstrating the existence of drug‐tolerant persister cell populations characterized by reversible growth arrest and stress‐adaptive programs [19, 32, 38, 39].

Taken together, these observations establish clonal selection and evolution as fundamental determinants of MRD biology. Within the trait framework, residual disease is best understood as the product of therapy‐driven evolution, in which genetic selection, epigenetic plasticity, and adaptive cellular states converge to generate a population of cells optimized for survival under stress. This evolutionary perspective provides a mechanistic foundation for the persistence of MRD and links naturally to additional layers of the trait model, including metabolic adaptation, immune escape, and microenvironmental interactions.

## 5 Epigenetic and Transcriptional Plasticity

While clonal selection provides a genetic framework for the emergence of MRD, it does not fully account for the adaptive flexibility observed in residual tumor populations. Increasing evidence indicates that MRD cells frequently exploit non‐genetic mechanisms, including epigenetic remodeling and transcriptional reprogramming, to survive therapeutic stress. These processes enable rapid phenotypic adaptation without requiring stable genetic alterations, thereby complementing and, in some cases, preceding clonal evolution [11, 12, 13, 26].

A key concept underlying this behavior is the existence of drug‐tolerant persister (DTP) states, first described as small subpopulations of cancer cells capable of surviving high concentrations of targeted therapies through reversible chromatin‐mediated mechanisms [19]. These cells are characterized by distinct epigenetic configurations, altered histone modifications, and transcriptional programs that promote survival under stress while maintaining the capacity to re‐enter proliferative states upon drug withdrawal. Importantly, DTP states are not fixed but represent dynamic and reversible phenotypes, highlighting the plastic nature of residual disease.

Subsequent studies across both solid tumors and hematologic malignancies have expanded this concept, demonstrating that epigenetic plasticity represents a broader mechanism of therapeutic resistance in cancer biology. Global chromatin reorganization can enable tumor cells to transition between transcriptional states associated with sensitivity and resistance, effectively buffering the impact of therapeutic pressure [2, 25, 37, 39]. These transitions may involve changes in enhancer landscapes, transcription factor networks, and lineage‐specific gene expression programs, allowing cells to adopt phenotypes that are better suited to survive in the presence of therapy.

In hematologic malignancies, epigenetic dysregulation is a defining feature of disease biology and plays a central role in MRD persistence. In acute leukemias, alterations in chromatin modifiers and DNA methylation patterns contribute to the maintenance of leukemic stem cell–like populations with enhanced resistance to chemotherapy [7, 40]. In MM, residual tumor cells have been shown to display transcriptional programs associated with stress adaptation, unfolded protein response, and survival signaling, consistent with a plastic and adaptive phenotype [6, 30].

A particularly important manifestation of transcriptional plasticity is lineage flexibility, whereby tumor cells transiently adopt alternative differentiation states that confer therapeutic resistance. This phenomenon, well‐established in solid tumors, is increasingly supported in hematologic malignancies, where shifts in differentiation state or stemness programs can modulate sensitivity to treatment and contribute to disease persistence and may underlie the functional heterogeneity observed within MRD populations [26, 37]. This capacity for phenotypic reprogramming further decouples resistance from fixed genetic alterations and underscores the adaptive potential of MRD populations.

Notably, epigenetic and transcriptional plasticity offer a mechanistic explanation for why certain resistant states can be reversed. Unlike genetically encoded resistance, which tends to be stable, non‐genetic adaptations can be temporary and dependent on context, enabling MRD cells to switch between drug‐tolerant and drug‐sensitive states. This fluctuating behavior adds to intratumoral heterogeneity and makes interpreting MRD status more complicated, as the biological potential of residual cells might not be fully reflected by static assessments. From a conceptual standpoint, these observations reinforce the notion that MRD is not defined solely by the presence of residual cells, but by their capacity to dynamically reconfigure their functional state in response to environmental and therapeutic cues. Epigenetic plasticity thus represents a core component of the MRD “trait,” enabling persistence in the absence of overt genetic resistance and providing a substrate for subsequent evolutionary selection.

The interplay between genetic selection and non‐genetic adaptation suggests that MRD should be viewed as a layered and integrative phenomenon, in which stable clonal architecture and reversible phenotypic states coexist and interact. Recent integrative single‐cell and multi‐omic studies further support this model, demonstrating that cell identity, epigenetic state, and clonal architecture are tightly coupled determinants of therapeutic response and relapse behavior [30]. This integrated perspective provides a more comprehensive understanding of disease persistence and highlights epigenetic regulation as a key therapeutic vulnerability in MRD‐directed strategies.

## 6 Metabolic Adaptation and Cellular Fitness

Beyond genetic selection and epigenetic plasticity, the persistence of MRD is critically supported by metabolic adaptation, which enables tumor cells to maintain viability under conditions of therapeutic and microenvironmental stress. Residual cells frequently adopt metabolic states that are distinct from those of the bulk tumor, favoring energy‐efficient and stress‐resilient programs over the highly proliferative, anabolic metabolism characteristic of untreated disease.

A recurrent feature of therapy‐persistent cells is a shift toward oxidative phosphorylation (OXPHOS) as a primary energy source. In contrast to glycolysis‐driven metabolism, which supports rapid proliferation, OXPHOS provides a more efficient means of ATP generation and is associated with enhanced resistance to metabolic stress. In AML, leukemia stem cells and chemoresistant populations have been shown to depend on mitochondrial respiration for survival, rendering them selectively vulnerable to OXPHOS inhibition [20, 22, 41]. Similar metabolic dependencies have been observed in MM, where residual disease is enriched for cells with increased mitochondrial activity and altered bioenergetic profiles [35, 42].

In addition to enhanced mitochondrial function, MRD cells often exhibit reduced anabolic activity and proliferative signaling, consistent with a state of metabolic quiescence. This adaptation allows cells to minimize energy expenditure and avoid the cytotoxic effects of therapies targeting rapidly dividing populations. Evidence from both hematologic malignancies and solid tumor systems indicates that quiescent cancer cells may rely on alternative metabolic pathways, including fatty acid oxidation and autophagy, to sustain survival under nutrient‐limited conditions [43, 44]. These findings suggest that metabolic reprogramming is not merely a consequence of reduced proliferation but an active survival strategy [20, 21, 31].

The tumor microenvironment further shapes metabolic adaptation in MRD. Residual cells often reside in hypoxic or nutrient‐deprived niches, such as the bone marrow microenvironment, where oxygen tension, stromal interactions, and cytokine signaling influence cellular metabolism. Hypoxia‐inducible pathways can promote metabolic flexibility, enabling cells to switch between glycolysis and oxidative metabolism as needed, whereas also contributing to resistance to apoptosis and therapy [28, 45]. In MM and leukemia, interactions with stromal cells have been shown to modulate metabolic pathways and enhance resistance to therapy, further supporting the role of the microenvironment in sustaining MRD [28].

Importantly, metabolic adaptation is closely linked to other features of the MRD phenotype, including quiescence, stemness, and stress tolerance. Mitochondrial function has been implicated in the maintenance of stem‐like properties in cancer cells, whereas reactive oxygen species (ROS) signaling can influence both survival and differentiation states. These interconnected pathways suggest that metabolic fitness is an integral component of the MRD trait, rather than an isolated adaptation.

From a therapeutic perspective, the metabolic dependencies of MRD cells represent a potential vulnerability. Preclinical and clinical studies have demonstrated that targeting mitochondrial metabolism, redox balance, or nutrient utilization can selectively affect therapy‐resistant populations, including leukemia stem cells and other MRD‐enriched compartments [41, 44]. More recent translational efforts further support combining metabolic inhibitors with standard or targeted therapies to eradicate persistent disease reservoirs [20, 22]. These approaches highlight the possibility of functionally targeting MRD based on metabolic traits, rather than relying solely on conventional cytotoxic strategies.

Collectively, these observations support the view that metabolic rewiring is a central determinant of MRD biology. Residual disease is characterized not only by reduced tumor burden but by the emergence of cells with enhanced bioenergetic efficiency, stress resistance, and adaptive flexibility. This metabolic phenotype contributes to the persistence of MRD and reinforces the concept of residual disease as a biologically defined trait shaped by functional fitness rather than cell number alone.

## 7 Immune Evasion and Residual Disease

An essential feature of MRD is the ability of residual tumor cells to evade immune surveillance, thereby escaping a major physiological mechanism of tumor control. Although cytotoxic therapies impose strong selective pressures on tumor‐intrinsic pathways, the immune system represents a parallel axis of selection that shapes the composition and behavior of residual disease. Within this context, MRD can be conceptualized as a state of dynamic equilibrium between tumor persistence and immune containment, in which residual cells survive despite ongoing immunologic pressure [23, 24, 46, 47].

Multiple mechanisms contribute to immune evasion in MRD. A central strategy involves the downregulation of antigen presentation machinery, including major histocompatibility complex (MHC) molecules, which reduces the visibility of tumor cells to cytotoxic T lymphocytes. Defects in antigen processing and presentation have been associated with resistance to immune‐mediated clearance across hematologic malignancies [48]. In parallel, tumor cells may upregulate immune checkpoint molecules, such as PD‐L1, which inhibit T‐cell activation and promote an immunosuppressive microenvironment. These mechanisms are not limited to advanced disease but can be detected in residual tumor populations, suggesting that immune escape is an early and integral component of MRD biology.

The tumor microenvironment further reinforces immune evasion through the establishment of immunosuppressive niches. Interactions with stromal cells, regulatory T cells, myeloid‐derived suppressor cells, and tumor‐associated macrophages can create a local environment that inhibits effective anti‐tumor immunity. In lymphoid malignancies, including DLBCL, alterations in the immune microenvironment have been linked to treatment resistance and relapse, with specific gene expression signatures reflecting immune exclusion or dysfunction [31, 49, 50]. These findings underscore the role of the immune contexture in determining the fate of residual disease.

Importantly, MRD may persist in a state of immune equilibrium, analogous to the equilibrium phase of cancer immunoediting, in which tumor cells are not fully eradicated but are held in check by immune responses [46, 47]. In this state, selective pressure from the immune system may further shape tumor evolution, favoring variants with reduced immunogenicity or enhanced resistance to immune effector mechanisms. Over time, these adaptations can culminate in immune escape, leading to overt clinical relapse.

Evidence supporting this model has emerged from studies of immunotherapy resistance. In both solid and hematologic malignancies, relapse following immune‐based therapies, such as checkpoint inhibitors or CAR T‐cell therapy, is often associated with loss of target antigens, defects in interferon signaling pathways, or alterations in antigen presentation [11, 51, 52, 53]. These findings provide direct evidence that immune‐mediated selection pressures can shape the residual disease compartment, further supporting the view of MRD as an adaptive phenotype.

From a conceptual standpoint, immune evasion highlights that MRD is not solely determined by tumor‐intrinsic properties but by the balance between tumor fitness and host immune competence. Two patients with similar levels of residual disease may experience divergent outcomes depending on the effectiveness of immune surveillance, the presence of immunosuppressive microenvironments, and the immunogenicity of the residual tumor population. This framework helps explain why MRD positivity does not uniformly translate into relapse and why some patients maintain long‐term disease control despite detectable residual disease.

Collectively, these observations establish immune evasion as a central component of the MRD “trait.” Residual disease reflects not only the survival of tumor cells under therapeutic pressure but also their capacity to coexist with, adapt to, and ultimately evade the immune system. Integrating immune dynamics into the understanding of MRD provides a more comprehensive framework for interpreting residual disease and highlights immune‐targeted strategies as critical components of MRD‐directed therapy.

## 8 Microenvironmental Niches and Spatial Protection

MRD is rarely a homogeneous population uniformly dispersed throughout the body. Instead, residual malignant cells preferentially localize within specialized microenvironmental niches that provide both physical and biochemical protection from therapeutic and immunologic pressures. In hematologic malignancies, the bone marrow microenvironment represents a primary sanctuary for MRD, offering structural support, stromal‐derived survival signals, and interactions with extracellular matrix components. Similarly, lymphoid stromal compartments in lymphomas and immune‐privileged sites, such as the central nervous system in acute lymphoblastic leukemia, constitute spatial refuges in which cytotoxic therapies and immune surveillance are less effective [21, 28, 54].

These niches confer multiple, non‐redundant layers of protection that enable long‐term persistence. They provide pro‐survival and anti‐apoptotic signals through paracrine cytokines, chemokines, and growth factors—including IL‐6, CXCL12, and BAFF—which activate intracellular pathways promoting resistance to therapy [28, 55, 56]. In parallel, the physical properties of these compartments—such as dense stromal architecture and limited vascular perfusion—can impair drug delivery, creating pharmacologic sanctuaries in which residual cells are exposed to sublethal drug concentrations [28].

Microenvironmental niches also actively shape immune responses, recruiting regulatory T cells and myeloid‐derived suppressor cells and producing immunosuppressive factors that attenuate cytotoxic T‐cell and natural killer cell activity. As a result, MRD cells may persist in a state of functional immune equilibrium, surviving immune‐mediated pressure while retaining the capacity for reactivation and expansion [46, 51, 57].

The spatial compartmentalization of MRD has direct clinical consequences. It contributes to heterogeneous detectability, as disease confined to discrete niches may evade conventional sampling, and to treatment resistance, as niche‐resident cells are less exposed to both cytotoxic agents and immune effectors. More broadly, spatial localization underscores that MRD is a context‐dependent phenotype, in which persistence is determined not only by intrinsic cellular properties but also by extrinsic environmental support and spatial constraints. Recent advances in spatial transcriptomics and single‐cell profiling have further demonstrated that MRD‐associated niches are highly organized ecosystems, characterized by defined cellular neighborhoods and signaling gradients that regulate tumor cell behavior and therapeutic response [7, 21, 30].

**FIGURE 2:**
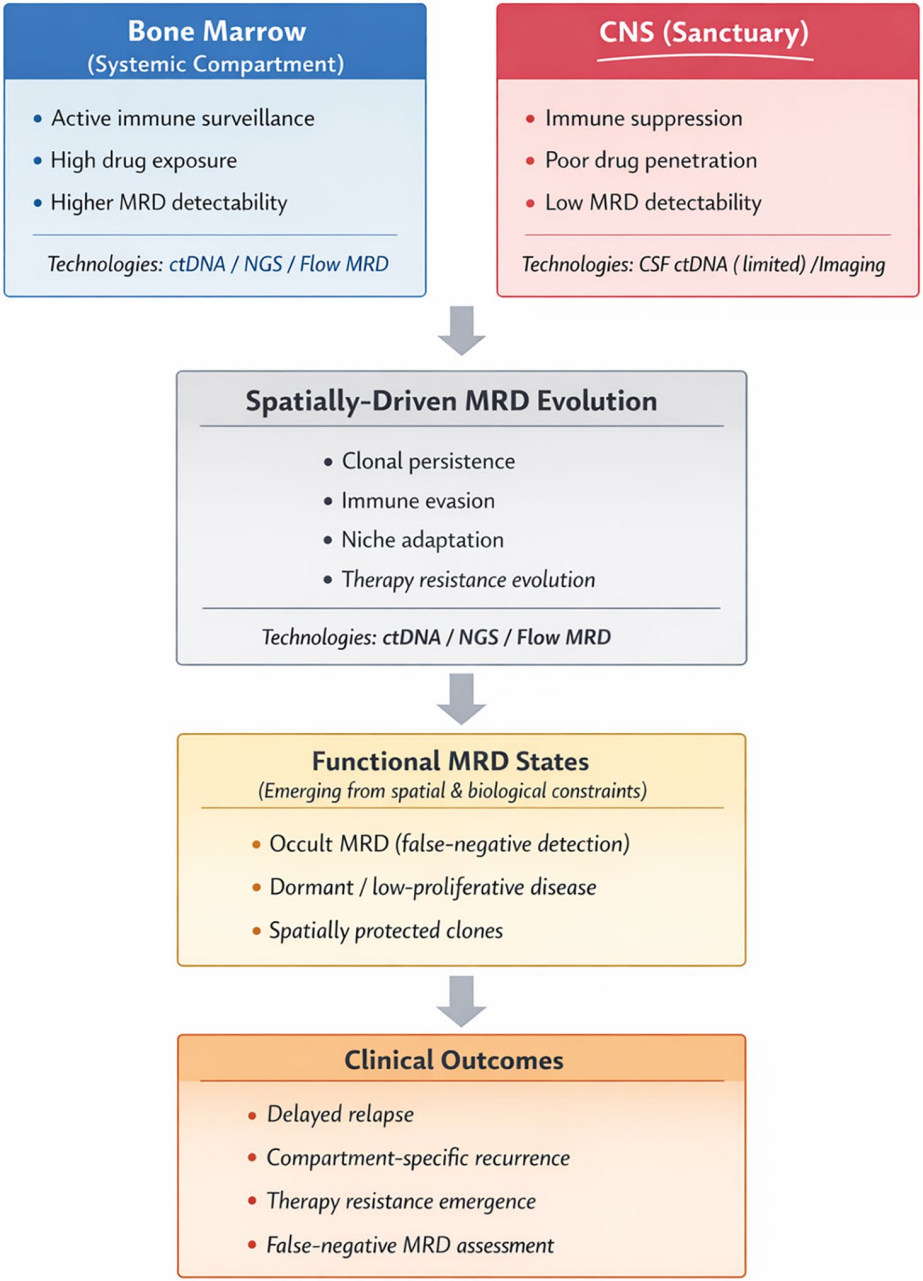
Biological determinants of minimal residual disease persistence and relapse. Schematic representation of the key biological and spatial mechanisms that govern minimal residual disease (MRD) as a dynamic and adaptive tumor phenotype in hematologic malignancies. MRD persistence is shaped by the interplay of clonal evolution, epigenetic and transcriptional plasticity, metabolic adaptation, immune evasion, and microenvironmental niche interactions. These processes are further modulated by spatial and compartment‐specific constraints, including differences in immune surveillance, drug penetration, and disease detectability across anatomical niches, such as bone marrow and sanctuary sites, including the central nervous system. Together, these biological and spatial pressures drive spatially structured MRD evolution, characterized by clonal persistence, therapy‐driven selection, immune escape, and niche adaptation. These biological and spatial features can be interrogated using emerging technologies, including circulating tumor DNA (ctDNA) analysis, single‐cell and epigenomic profiling, metabolic imaging, immune phenotyping, and spatial transcriptomics, enabling refined characterization of residual disease states. This evolutionary process gives rise to distinct functional MRD states, including dormant or low‐proliferative disease, occult MRD with false‐negative detectability, and spatially protected residual clones. Collectively, these states determine clinically relevant outcomes, including delayed relapse, compartment‐specific recurrence, false‐negative MRD assessment, and therapeutic resistance. This framework emphasizes MRD as a spatially organized and biologically adaptive system rather than a static residual tumor burden. Figure generation assisted by AI‐based tools.

The integration of spatial protection with evolutionary selection, cellular plasticity, metabolic adaptation, and immune evasion defines MRD as a multidimensional biological trait, rather than a simple measure of residual tumor burden. These mechanisms collectively define the biological basis of MRD persistence and their therapeutic implications are summarized in Table 2. Moreover, these interacting mechanisms, and their spatial organization within protective niches, are schematically illustrated in Figure 2.

**TABLE 2:** Biological mechanisms of MRD persistence and therapeutic implications.

Mechanism	Biological features	Clinical implications	Potential therapeutic strategies
Clonal evolution and selection	Enrichment of pre‐existing resistant subclones; therapy‐driven genetic selection	Early relapse; emergence of resistant disease	Targeted therapies based on mutational profile; combination strategies to prevent clonal escape
Epigenetic and transcriptional plasticity	Chromatin remodeling; reversible drug‐tolerant states; lineage flexibility	Variable treatment response; transient resistance without new mutations	Epigenetic therapies (e.g., HDAC or DNMT inhibitors); strategies to disrupt adaptive transcriptional programs
Metabolic adaptation	Increased oxidative phosphorylation; reduced anabolic activity; adaptation to hypoxia and nutrient stress	Resistance to therapies targeting proliferating cells; long‐term persistence	Mitochondrial inhibitors; metabolic modulators; targeting autophagy or stress‐response pathways
Immune evasion	Downregulation of antigen presentation; upregulation of checkpoint molecules; immunosuppressive signaling	Failure of immune‐mediated clearance; risk of late relapse	Immune checkpoint inhibitors; CAR‐T cells; bispecific antibodies; immune microenvironment modulation
Microenvironmental niche protection	Interaction with stromal cells; cytokine‐mediated survival signals; physical protection from drugs	Sanctuary sites; reduced drug penetration; persistence despite systemic therapy	Agents targeting tumor–microenvironment interactions; niche disruption strategies; improved drug delivery
Cellular dormancy and quiescence	Reduced proliferation; reversible cell‐cycle arrest; survival under stress	Minimal sensitivity to cytotoxic therapies; delayed relapse	Therapies targeting dormant cells; induction of cell‐cycle re‐entry followed by cytotoxic treatment

## 9 Explaining Discordance: Molecular Versus Imaging MRD

Discordance between molecular and imaging‐based assessments of MRD is a well‐recognized phenomenon across hematologic malignancies and represents a critical challenge for disease monitoring and clinical decision‐making. Patients may demonstrate detectable ctDNA despite achieving complete metabolic response on PET, while others exhibit persistent PET positivity in the absence of molecular evidence of disease. Although such discrepancies are often attributed to differences in assay sensitivity, a purely technical explanation is insufficient. Instead, these observations reflect the fact that PET and ctDNA interrogate fundamentally distinct and complementary biological dimensions of residual disease.

ctDNA‐based assays provide a highly sensitive measure of systemic clonal persistence, detecting tumor‐derived genetic material released into the circulation and enabling longitudinal tracking of disease dynamics. In aggressive lymphomas, including DLBCL and Hodgkin lymphoma, serial ctDNA monitoring can identify molecular relapse months before clinical or radiographic progression [3, 13, 14]. In this context, ctDNA positivity in the setting of PET negativity reflects the presence of biologically active but spatially limited OR METABOLICALLY QUIESCENT disease, consistent with residual clones that persist below the resolution of imaging modalities.

In contrast, PET imaging—most commonly using 18F‐fluorodeoxyglucose (FDG)—captures metabolic activity and spatial localization of disease. FDG uptake reflects glucose metabolism, which is increased not only in malignant cells but also in activated immune cells and inflamed tissues. As a result, PET positivity does not necessarily equate to a viable or clonally expanding tumor. In lymphoma, particularly DLBCL, false‐positive PET findings are well documented and may arise from post‐treatment inflammation, fibrosis, or immune‐mediated processes [16].

These differences provide a mechanistic basis for the two most common discordant scenarios. MRD‐positive/PET‐negative disease reflects early molecular persistence, in which residual clones are detectable systemically but have not yet formed metabolically active or spatially consolidated lesions. This pattern has been consistently associated with increased risk of relapse in DLBCL, supporting the biological relevance of ctDNA as an early marker of disease re‐emergence [13, 15]. Conversely, MRD‐negative/PET‐positive findings often represent metabolically active but non‐malignant tissue, particularly in the post‐treatment setting, where immune activation and tissue remodeling contribute to FDG uptake. In such cases, the absence of ctDNA suggests a lack of clonally persistent disease and is frequently associated with favorable outcomes.

A similar conceptual framework applies to MM, although the modalities differ. Molecular MRD assessment using next‐generation sequencing or flow cytometry in bone marrow provides a highly sensitive measure of residual clonal plasma cells, whereas functional imaging with PET/CT identifies metabolically active focal lesions. Discordance between these approaches is increasingly recognized: patients may achieve bone marrow MRD negativity while retaining PET‐positive lesions, or conversely, demonstrate persistent molecular disease despite imaging remission. Studies have shown that combined and longitudinal assessment improves prognostic accuracy, as PET positivity identifies spatially localized disease not captured by bone marrow sampling, while molecular MRD detects diffuse or low‐level clonal persistence [4, 10, 58].

Importantly, spatial compartmentalization contributes significantly to discordance. As discussed in the previous section, residual disease may be confined to specific microenvironmental niches, leading to underrepresentation in circulating biomarkers or bone marrow samples while remaining detectable by imaging. Conversely, small, widely disseminated populations may release detectable ctDNA without forming discrete lesions. Temporal dynamics further amplify these differences: ctDNA may capture early clonal resurgence and molecular progression, whereas PET reflects later stages of metabolic activation and tumor expansion.

Within this framework, discordance between molecular and imaging MRD should not be interpreted as conflicting information but as evidence that MRD is a multidimensional biological entity. Each modality captures a distinct aspect of the residual disease phenotype—ctDNA reflecting clonal persistence and PET reflecting functional metabolic and spatial expression of disease.

These observations are consistent with the trait model of MRD. If residual disease were solely a function of tumor burden, concordance between modalities would be expected. The observed divergence instead indicates that biological properties—such as proliferative capacity, metabolic activity, and microenvironmental context—determine detectability and clinical behavior.

From a clinical perspective, integrating molecular and imaging assessments provides a more comprehensive evaluation of MRD, capturing both systemic and spatial dimensions of disease persistence. Such an approach may improve risk stratification and guide therapeutic decision‐making, particularly in settings where discordant findings identify patients at differential risk of relapse. Future MRD frameworks will likely require integrative, multimodal models combining ctDNA, imaging, immune profiling, and functional biomarkers to capture the complexity of residual disease biology fully.

## 10 Clinical Implications of a Phenotypic Model

Reframing MRD as a biologically defined phenotype rather than a purely quantitative state has important implications for clinical practice, influencing risk stratification, therapeutic decision‐making, and clinical trial design. By integrating tumor‐intrinsic properties with microenvironmental and immune context, the “trait” model provides a more nuanced, mechanistically grounded framework for interpreting MRD and guiding intervention strategies.

### 10.1 Risk Stratification

Within the conventional paradigm, MRD is often treated as a binary variable—positive or negative—implicitly assuming that all residual disease carries similar biological and clinical significance. However, accumulating evidence indicates that outcomes among MRD‐positive patients are highly heterogeneous, suggesting that the qualitative and functional features of residual cells play a critical role in determining relapse risk.

In MM, MRD negativity assessed by next‐generation sequencing or flow cytometry is strongly associated with improved progression‐free and overall survival, yet a subset of MRD‐negative patients still experience relapse, while some MRD‐positive patients maintain durable disease control [1, 4, 6]. Similarly, in lymphomas, ctDNA positivity following therapy identifies patients at increased risk of relapse, but the kinetics and magnitude of molecular persistence further refine prognostic stratification [3, 13, 15].

These observations support a model in which MRD should be interpreted within a broader biological context, incorporating factors such as clonal composition, evolutionary dynamics, growth kinetics, metabolic activity, immune competence, and spatial localization. Rather than a binary endpoint, MRD becomes a continuous, dynamic, and multidimensional variable, enabling more refined and individualized risk assessment. This heterogeneity is particularly evident in CLL, where MRD status must be interpreted in the context of treatment strategy: MRD‐positive patients may experience prolonged disease control under continuous targeted therapy, whereas in fixed‐duration regimens, MRD kinetics following treatment discontinuation more directly predict relapse risk.

### 10.2 Therapeutic Implications

Viewing MRD as a phenotypic trait implies that residual disease may require distinct therapeutic strategies compared with the bulk tumor. Conventional cytotoxic approaches, which primarily target rapidly proliferating cells, may be less effective against MRD populations characterized by quiescence, metabolic rewiring, and microenvironmental protection.

Targeting MRD therefore necessitates approaches directed at the specific biological vulnerabilities of residual cells. For example, therapies that disrupt survival signals within the bone marrow niche or interfere with CXCR4–CXCL12 signaling may enhance the elimination of niche‐protected cells [59, 60]. Similarly, agents targeting mitochondrial metabolism or oxidative phosphorylation have shown activity against therapy‐resistant populations, including leukemia stem cells [20, 22, 41].

Immune‐based strategies are particularly relevant in the MRD setting. Given the role of immune evasion in residual disease persistence, therapies such as monoclonal antibodies, bispecific T‐cell engagers, and CAR T cells may be especially effective when tumor burden is low and immune function is relatively preserved [51]. Importantly, the timing, sequencing, and biological matching of these therapies should be adapted to the specific MRD phenotype (e.g., immune‐cold vs. immune‐active, metabolically quiescent vs. proliferative states).

For example, in a patient with DLBCL achieving metabolic remission on PET, detection of ctDNA positivity could indicate persistent clonal disease. Integration with immune profiling showing reduced T‐cell activation and spatial analysis demonstrating residual niche localization could identify a biologically active MRD phenotype with elevated relapse potential. In such a scenario, MRD would not be interpreted solely as molecular positivity, but as a composite profile combining clonal persistence, immune escape, and spatial protection. This integrated interpretation could support early therapeutic intervention, such as immunotherapy or targeted consolidation, rather than observation alone.

In CLL, these dynamics are further modulated by targeted and immune‐based therapies, reinforcing the need to interpret MRD within a biologically integrated framework [61].

These considerations highlight that MRD‐directed therapy should not be based solely on the presence of residual disease, but on its biological architecture, including proliferative status, metabolic dependencies, immune interaction, and niche localization. This paradigm supports a shift toward MRD‐guided, biology‐adapted therapeutic strategies rather than uniform escalation or de‐escalation approaches.

### 10.3 Implications for Clinical Trial Design

The integration of MRD into clinical trials has already transformed drug development, with MRD negativity increasingly used as a surrogate endpoint. However, the phenotypic model suggests that current MRD endpoints may be overly reductionist, failing to capture the biological complexity of residual disease.

Future trial designs should move beyond binary MRD assessment to incorporate quantitative temporal and functional dimensions, including depth of response, kinetics of MRD clearance, and patterns of re‐emergence over time. Serial monitoring of ctDNA, for example, can provide dynamic information on clonal evolution and early relapse, while functional imaging can capture spatial and metabolic aspects of disease persistence [3, 4].

In addition, integrating biological correlates—such as immune profiling, metabolic signatures, and single‐cell analyses—may enable identification of distinct MRD phenotypes associated with differential therapeutic sensitivity [7]. This approach could support the development of adaptive and response‐adaptive trial designs, in which treatment is modified based on evolving MRD characteristics rather than fixed time points.

Finally, the recognition of MRD as a multidimensional trait supports the use of composite and integrative endpoints that combine molecular, imaging, and clinical parameters, providing a more comprehensive assessment of treatment efficacy. Such strategies may improve the predictive value of MRD and accelerate the development of therapies specifically targeting residual disease. Ultimately, this framework aligns MRD assessment with precision oncology, enabling biologically informed intervention at the stage of minimal disease burden.

## 11 Future Directions

The evolving understanding of MRD as a biological trait rather than a purely quantitative state necessitates a rethinking of how MRD is assessed, interpreted, and utilized in clinical practice. Traditional MRD evaluation, based on single‐point measurements of residual cell number, provides important prognostic information but may not fully capture the dynamic, multidimensional, and context‐dependent complexity of persistent disease. A next‐generation MRD framework should integrate molecular, cellular, spatial, immune, and functional dimensions to generate a more comprehensive and predictive model of residual disease in hematologic malignancies.

To translate the proposed biological framework into measurable parameters, each MRD axis can be associated with emerging biomarkers and analytical technologies that capture distinct functional dimensions of residual disease: clonal fitness may be assessed through longitudinal genomic profiling using NGS or ctDNA, enabling the detection of resistant subclones, clonal dynamics, and evolutionary trajectories during therapy; phenotypic plasticity can be explored through single‐cell RNA sequencing, chromatin accessibility assays (e.g., ATAC‐seq), and epigenetic profiling, which reveal transcriptional state transitions and drug‐tolerant persister phenotypes within residual populations; metabolic adaptability may be characterized using metabolic imaging, mitochondrial activity assays, and transcriptomic signatures associated with oxidative phosphorylation or fatty acid oxidation, which have been linked to therapy‐resistant cell states; immune evasion can be evaluated through immune profiling approaches, including multiplex immunophenotyping, single‐cell immune analysis, and assessment of checkpoint molecule expression (e.g., PD‐L1), providing insight into tumor–immune interactions during MRD; microenvironmental dependence may be captured through spatial transcriptomics, spatial proteomics, and advanced imaging techniques that identify niche‐localized tumor cells and their interactions with stromal and immune components.

This multidimensional characterization allows MRD to be interpreted not solely as residual tumor burden but as a composite biological phenotype reflecting the functional capacity of residual cells to persist and re‐expand.

**FIGURE 3:**
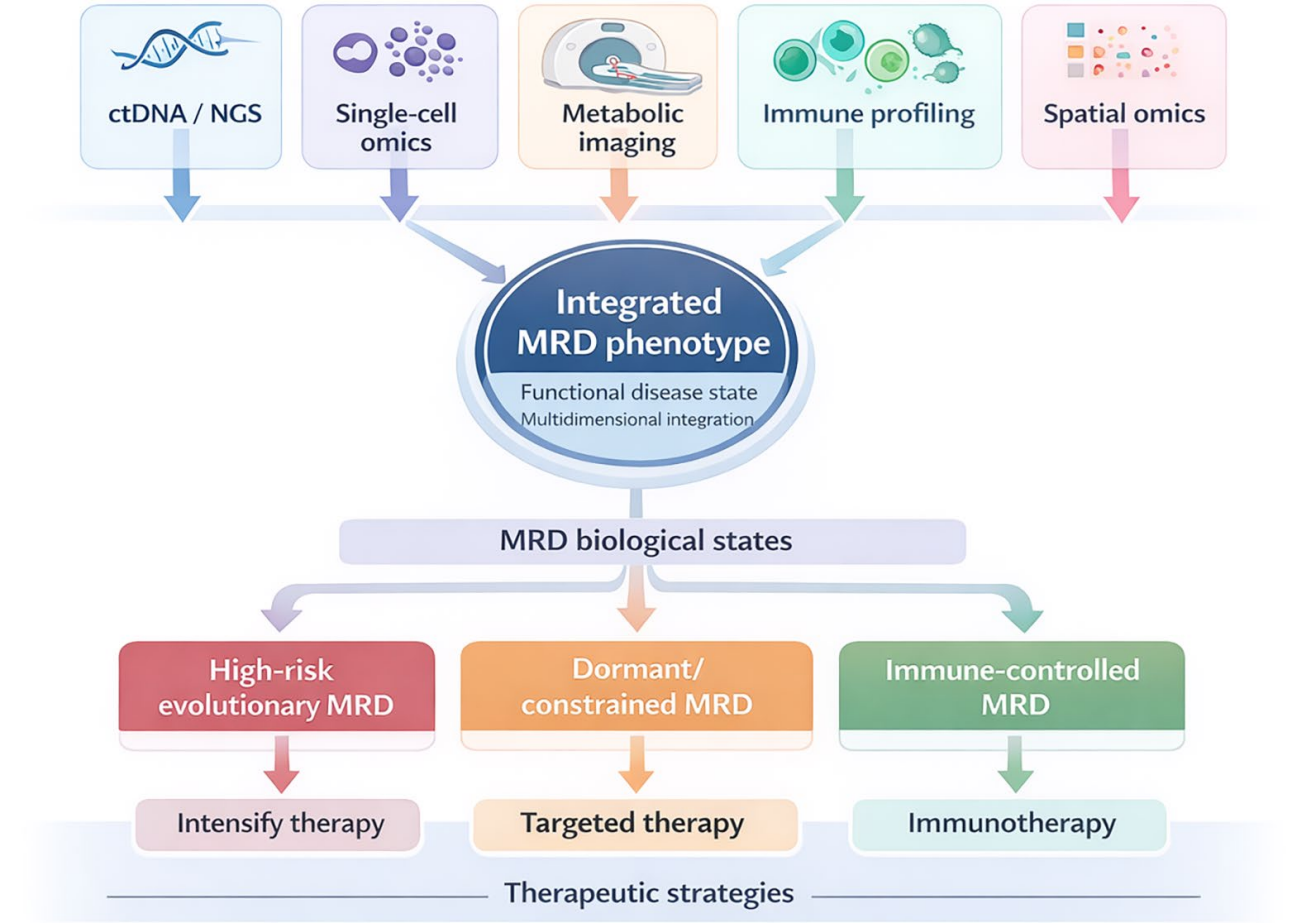
Multidimensional framework for minimal residual disease assessment. Schematic representation of a next‐generation, multidimensional approach to minimal residual disease (MRD) evaluation in hematologic malignancies. The model integrates complementary biological and technological domains, including molecular burden (e.g., circulating tumor DNA and next‐generation sequencing), functional imaging (e.g., PET/CT and MRI), immune profiling (e.g., immune cell composition and checkpoint expression), and single‐cell and spatial analyses, which together capture the heterogeneity and biological context of residual disease. In this framework, these multimodal data streams are integrated into a unified multidimensional MRD assessment, which generates an integrated MRD phenotype reflecting the functional state of residual disease beyond simple tumor burden. This integration enables stratification of MRD into biologically distinct states with different clinical behaviors, including high‐risk evolutionary disease, dormant or constrained residual populations, and immune‐controlled MRD. Clinically, this framework supports improved risk stratification, biology‐adapted therapeutic targeting, and the development of clinical trials incorporating dynamic, multimodal, and biologically informed MRD endpoints. Overall, it provides an operational strategy for translating multidimensional biological data into clinically actionable MRD‐based decision‐making. Figure generation assisted by AI‐based tools.

A schematic representation of this multidimensional framework is provided in Figure 3.

At the molecular level, high‐sensitivity approaches such as ctDNA, NGS, and targeted MRD assays enable quantification of residual clones and longitudinal tracking of clonal evolution. Serial molecular profiling allows early detection of emerging resistant subclones, assessment of intratumoral heterogeneity, and monitoring of therapy‐induced selective pressures, thereby providing real‐time, dynamic information on disease fitness and evolutionary trajectories, extending beyond static MRD positivity or negativity [13, 62, 63, 64].

Functional imaging modalities, including PET/CT and advanced MRI techniques, complement molecular approaches by providing spatial and metabolic context. These tools can identify sanctuary sites and regions of residual metabolic activity that may be underrepresented by molecular assays, particularly in anatomically compartmentalized disease. Integration of imaging and molecular data is especially valuable in resolving discordant findings, where molecular positivity is not accompanied by radiographic abnormalities, or imaging lesions reflect non‐viable or inflammatory tissue rather than active disease [16, 30], particularly in anatomically compartmentalized disease [14].

In parallel, immune profiling provides critical insight into the interaction between residual tumor cells and host immunity. Characterization of immune cell composition, checkpoint expression, and microenvironmental immunosuppression may help identify mechanisms of immune escape and inform the use of immunotherapeutic strategies, including bispecific antibodies and CAR T‐cell therapies, in the MRD setting [65, 66, 67]. Advances in single‐cell and spatial omics technologies further expand the resolution of MRD assessment. Comparative studies, such as CPX‐351 versus venetoclax plus hypomethylating agents, illustrate the importance of integrating therapy‐specific effects into MRD evaluation [68]. These approaches enable detailed characterization of transcriptional states, epigenetic programs, metabolic adaptations, and microenvironmental interactions at the level of individual cells, whereas preserving spatial context. Such analyses can identify rare, therapy‐resistant populations and map their localization within protective niches [7, 21, 57, 69].

Beyond technological integration, future MRD frameworks will require computational and systems‐level approaches capable of integrating multimodal datasets into clinically actionable models. Machine learning and artificial intelligence–based strategies are increasingly being applied to combine molecular, imaging, and clinical variables, enabling prediction of relapse risk and identification of biologically distinct MRD phenotypes [35].

Although this framework is developed in the context of hematologic malignancies, similar principles may extend to solid tumors, where ctDNA and other liquid biopsy approaches are increasingly used to monitor minimal disease. Current evidence indicates that ctDNA‐based MRD detection can predict relapse and guide post‐treatment interventions in multiple solid tumor types, including colorectal, lung, and breast cancers, and may reflect underlying clonal evolution, spatial heterogeneity, and microenvironmental interactions [70, 71, 72, 73]. Future studies should determine to what extent shared axes—such as clonal evolution, cellular plasticity, metabolic adaptation, immune escape, and spatial niche protection—define MRD across tumor types, and how disease‐specific microenvironments modulate these processes.

Ultimately, integrating these complementary dimensions may transform MRD assessment from a single‐parameter measurement into a multilayered, systems‐level disease model that captures both the quantity and biological quality and functional competence of residual cells. Such an approach has the potential to improve relapse prediction, guide adaptive therapeutic strategies, and support the development of clinical trials targeting the specific vulnerabilities of residual disease. The principal technologies for MRD assessment, together with their respective strengths and limitations, are summarized in Table 3.

**TABLE 3:** Technologies for minimal residual disease assessment: Characteristics, strengths, and limitations.

Method	Sensitivity (approx.)	Biological information	Advantages	Limitations	Clinical applications
Multiparameter flow cytometry (MFC)	10^−4^–10^−5^	Immunophenotypic residual cells	Widely available; rapid; standardized in several diseases	Operator‐dependent; limited sensitivity vs. NGS; requires bone marrow	Routine MRD assessment (e.g., multiple myeloma, ALL, AML)
Next‐generation sequencing (NGS)	10^−5^–10^−6^	Clonal sequences (Ig/TCR or mutation‐based)	High sensitivity; reproducible; standardized platforms	Cost; longer turnaround; requires baseline clonotype identification	Deep MRD assessment; clinical trials; response evaluation
Circulating tumor DNA (ctDNA)	Variable (10^−4^–10^−6^)	Tumor‐derived DNA in plasma; clonal evolution	Non‐invasive; enables longitudinal monitoring; captures heterogeneity	Limited spatial resolution; variable sensitivity; requires assay design	Monitoring in lymphomas and myeloma; early relapse detection
Digital PCR (dPCR)	10^−4^–10^−5^	Specific mutations or rearrangements	High precision; rapid; cost‐effective for known targets	Limited multiplexing; requires known mutation	Targeted MRD tracking in selected cases
Imaging (PET/CT)	~10^−2^–10^−3^ (low sensitivity)	Metabolic activity and spatial distribution	Whole‐body assessment; detects extramedullary disease	Low sensitivity for minimal disease; false positives (inflammation)	Lymphomas; assessment of residual masses; complementary to molecular MRD
Magnetic resonance imaging (MRI)	Low–moderate	Structural and functional tissue changes	High spatial resolution; no radiation; useful in bone disease	Limited sensitivity for MRD; not disease‐specific	Myeloma bone disease; CNS involvement
Single‐cell and spatial omics (emerging)	Ultra‐high (research level)	Cellular heterogeneity; transcriptional and spatial context	High‐resolution biological insight; identifies resistant subpopulations	Limited clinical availability; high cost; complex analysis	Research; future MRD characterization and precision medicine

In this perspective, MRD assessment evolves from a static biomarker into an integrative platform for precision oncology, enabling biologically informed intervention at the stage of minimal disease burden and redefining therapeutic goals from disease reduction to durable disease control or eradication.

Beyond these research priorities, this conceptual framework has immediate implications for clinical interpretation and therapeutic strategies.

## 12 Clinical and Translational Implications

Conceptualizing MRD as a biological trait has several important clinical implications. First, MRD negativity may not uniformly indicate the absence of clinically relevant disease, as biologically competent residual cells may persist below detection thresholds. Second, MRD positivity does not necessarily predict relapse, as residual cells may exist in biologically constrained or immune‐controlled states.

Third, MRD should be interpreted as a dynamic process rather than a static measurement, reflecting evolving interactions between tumor cells, therapy, and the host environment. Fourth, discordance between molecular and imaging‐based assessments should be understood as evidence that different modalities capture complementary aspects of residual disease biology.

Finally, MRD‐directed therapeutic strategies should aim not only to reduce tumor burden but also to target the biological mechanisms that sustain residual disease, including metabolic adaptation, immune evasion, and microenvironmental protection.

## 13 Conclusions

MRD has traditionally been conceptualized as a quantitative state reflecting low levels of tumor burden below the limits of conventional detection. Although this framework has enabled major advances in response assessment, prognostication, and clinical trial design, it does not fully capture the dynamic and biologically heterogeneous complexity of disease persistence. This perspective supports interpreting MRD as a biologically informed phenotype of persistence [8, 19, 22].

This reconceptualization has important implications. It explains key clinical observations that remain unresolved within a purely quantitative paradigm, including heterogeneous clinical trajectories among MRD‐positive patients, long‐term disease control despite detectable residual disease, and discordance between molecular and imaging‐based assessments. In this context, MRD reflects not simply the number of residual cells but their functional competence and evolutionary potential to survive, adapt, and eventually re‐expand under selective pressure.

Recognizing MRD as a “trait” rather than a “state” supports a shift toward integrative and multidimensional assessment strategies that integrate molecular burden, spatial localization, immune context, and cellular fitness. Such an approach provides a more accurate framework for risk stratification and offers a rationale for developing biology‐adapted, MRD‐directed therapeutic strategies targeting the specific vulnerabilities of residual disease. In MM, this paradigm shift aligns with evolving concepts of functional cure, in which sustained disease control may be achieved despite low‐level persistence [74, 75, 76, 77].

Ultimately, advancing MRD from a static measurement to a dynamic, systems‐level, and biologically informed disease model has the potential to improve relapse prediction, refine treatment personalization, and accelerate the development of MRD‐directed therapies. The implementation of this paradigm shift—through multimodal MRD assessment, adaptive clinical trial design, and mechanism‐based therapeutic targeting—will be essential for translating mechanistic insights into meaningful clinical benefit across hematologic malignancies.

In this perspective, MRD is no longer merely a marker of residual disease, but a window into the biology of persistence—providing an opportunity to intervene at the critical interface between remission and relapse.

## Author Contributions

All authors contributed to the manuscript and were involved in revisions and proofreading. All authors approved the submitted version.

## Funding

The authors have nothing to report.

## Ethics Statement

The authors have nothing to report.

## Conflicts of Interest

The authors declare no conflicts of interest.

## Data Availability

Data sharing not applicable to this article as no datasets were generated or analysed during the current study.
